# Street Choice Logit Model for Visitors in Shopping Districts

**DOI:** 10.3390/bs4030154

**Published:** 2014-07-04

**Authors:** Ko Kawada, Takashi Yamada, Tatsuya Kishimoto

**Affiliations:** 1 Graduate School of Science and Technology, Keio University, Yokohama 223-8522, Japan; E-Mails: fptbp372@a7.keio.jp (K.K.); takashi-yamada@a2.keio.jp (T.Y.); 2 Department of System Design Engineering, Faculty of Science and Technology, Keio University, Yokohama 223-8522, Japan

**Keywords:** pedestrian distribution, pedestrian behavior, street choice, route choice, shopping district, strolling visitors, logit model, space syntax

## Abstract

In this study, we propose two models for predicting people’s activity. The first model is the pedestrian distribution prediction (or postdiction) model by multiple regression analysis using space syntax indices of urban fabric and people distribution data obtained from a field survey. The second model is a street choice model for visitors using multinomial logit model. We performed a questionnaire survey on the field to investigate the strolling routes of 46 visitors and obtained a total of 1211 street choices in their routes. We proposed a utility function, sum of weighted space syntax indices, and other indices, and estimated the parameters for weights on the basis of maximum likelihood. These models consider both street networks, distance from destination, direction of the street choice and other spatial compositions (numbers of pedestrians, cars, shops, and elevation). The first model explains the characteristics of the street where many people tend to walk or stay. The second model explains the mechanism underlying the street choice of visitors and clarifies the differences in the weights of street choice parameters among the various attributes, such as gender, existence of destinations, number of people, *etc.* For all the attributes considered, the influences of DISTANCE and DIRECTION are strong. On the other hand, the influences of Int.V, SHOPS, CARS, ELEVATION, and WIDTH are different for each attribute. People with defined destinations tend to choose streets that “have more shops, and are wider and lower”. In contrast, people with undefined destinations tend to choose streets of high Int.V. The choice of males is affected by Int.V, SHOPS, WIDTH (positive) and CARS (negative). Females prefer streets that have many shops, and couples tend to choose downhill streets. The behavior of individual persons is affected by all variables. The behavior of people visiting in groups is affected by SHOP and WIDTH (positive).

## 1. Introduction

In recent years, the importance of realizing the cities in which people can live without dependence on cars has been recognized. Therefore, there have been concerns about the need to create user-friendly cities that are equipped with specialized infrastructure. To create such cities, it is necessary that streets in cities are designed to be not only effective pathways for movement but also as attractive spaces that are ideal for a stroll because pedestrians enhance the liveliness of a city. Accordingly, to enable successful urban planning, we need to examine the characteristics of streets on which a large number of people stroll. We propose two models for predicting pedestrian activity. First model is a pedestrian distribution model. This model shows the characteristics of streets where many people stroll. Second model is a street choice model. This model shows the characteristics of street which people tend to choose when they stroll. This model explains pedestrian activity quantitatively. Street choice may be different by people’s personal character; therefore we examine the second model by the attribute of the stroller such as genders, ages, and so on. In the future, combining these models, it would be possible to estimate the distribution of pedestrians by the estimation of strolling route based on the composition of peoples’ age, gender, or occupation.

There are many former related researches about the route choice and strolling behavior in commercial district or shopping center. Gil, Lemilij, Rose, and Penn [1] clarified that distinct clusters of shopping strategy can be defined in terms of characteristic search trails through a store and that these trails correlated with specific shopper profiles. Millonig and Schechtner [2], on the basis of the assessment of shoppers, revealed that motion pattern and preferences tend to differ depending on their profile. Tsukaguchi and Matsuda [3] found that pedestrians’ direction of movement changes depending on the angle formed between the street and a straight line drawn between the destination and the present location.

Furthermore, Golledge [4,5] investigated the route choice by considering the cognitive map and distance, and revealed the criteria for selecting a particular route to reach the destination. Takegami and Tusgaguchi [6] created a pedestrian route choice model considering the locations of destinations and the direction of movement. Kneidl and Borrmann [7] modeled the route choice taking into consideration the familiarity of the pedestrian with the spaces between the origin and the destination. Sakurai and Yoshizuka [8] formulated a grid street model and estimated the number of pedestrians by using the pedestrian survey data. Zhu and Timmermans [9] comprehended the overall shopping behavior on the basis of heuristic models, which involves the route choice model using principles of bounded rationality under the circumstances of incomplete information. Sueshige and Morozumi [10] considered the effects of changes in visual information for pedestrians by a linked QTVR (QuickTime Virtual Reality) simulator. Oiwa, Misaka and Kaneda [11] analyzed the dynamics of the behaviors of both shops and visitors by using data from two surveys performed in 1998 and in 2003 in Nagoya. Kawanabe and Kawashima [12] clarified the effects of spatial compositions for people who use trams. Matsumoto and Funabiki [13] revealed the relationship between the occurrences of staying and space conditions in an underground shopping arcade, such as advertisements and information displays.

Previous studies in this regard considered two aspects. Some studies focused on spatial connections such as street networks, while the others focused on spatial compositions such as the number of shops and visual elements. However, we assumed that pedestrians perceived both space connections and compositions while strolling. Therefore, this paper suggests two models that consider both space connections and compositions. We used seven variables in these models. Space connections included the following variables: (1) integration value obtained from the space syntax and (2) the shortest distance to a destination (or a station). Space compositions included the following: (3) number of pedestrians, (4) cars, (5) shops, (6) width of streets, and (7) altitude. In order to obtain the number of pedestrians and cars, we performed a field survey. One is a pedestrian distribution model that explains the relationship between pedestrian distribution and spaces. Using this model, we aim to gain deeper insights on the distribution characteristics of visitors on the basis of the spatial connection and composition. This model was developed using multiple regression analysis. The other model is a street choice model that explains the influence of elements when people choose a route. This was developed using logit model based on routes obtained from a questionnaire survey. Logit model is a popular model in transportation science, which has been used for the prediction of mode choice (Hensher, Rose, and Greene [14] and Japan Society of Civil Engineers [15]). Through the analysis of street choices using the multinomial logit model, we clarify the utility function of street choice. Furthermore, we analyze the difference in the street choice of the visitors by comparing the parameters of different street choices depending on the visitors’ profiles and whether or not the visitors have a defined destination.

## 2. Method

### 2.1. Study Area

**Figure 1:**
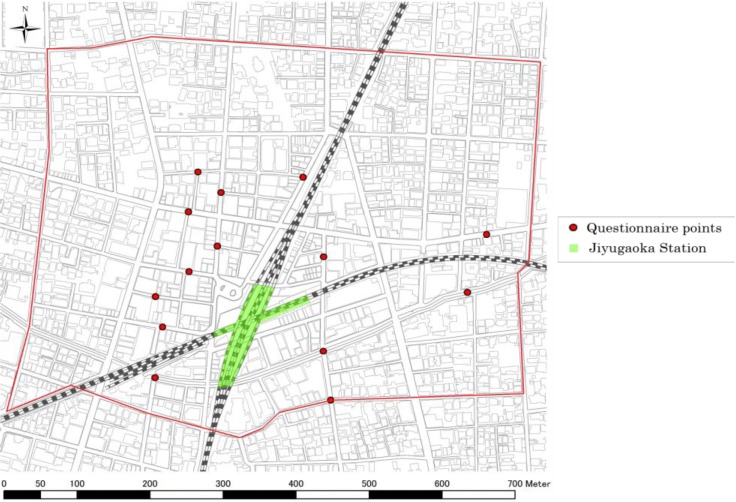
Study area and questionnaire survey points.

The area of focus in this study is around the Jiyugaoka Station in the suburbs of Tokyo (Figure 1), around which many shops are distributed. Meanwhile, there are intricate connections between the streets. Therefore, we expect that visitors’ strolling routes in this area would be more complicated and different from those on a straight shopping street. It is assumed that visitors who exit the station stroll within this area because a sufficient number of walkers can be considered for the modeling, and it contains a large number of shops, while outside this area, the number of walkers decreases significantly. In this study, we made “a segment map” of the study area, which is prepared by Depthmap. A “segment” indicates the space between two adjacent intersections. The number of segments is 789.

### 2.2. Variables of Two Models

We used eight variables for modeling, namely “the logarithm of density of pedestrians on each segment (PEDESTRIANS)”, “number of cars on each segment per 1 m (CARS)”, “number of shops facing each segment per 1 m (SHOPS)”, “elevation of each node (ELEVATION)”, “integration value (Int.V)”, “distance from a specific place (DISTANCE)”, “width of each street (WIDTH)”, and “direction of street choice (DIRECTION)” (Table 1).

**Table 1:** Variables and how they are determined.

Variables	Method of data collection
PEDESTRIANS	Field survey
CARS	
SHOPS	Town Pages (NTT yellow pages)
ELEVATION	Digital map 5m mesh (elevation)
DISTANCE	A program using Dijkstra’s algorithm
Int.V	Space Syntax
WIDTH	Measuring result (field survey)
DIRECTION	questionnaire survey (0 or 1)

NTT: Nippon Telegraph and Telephone Corporation.

In the distribution model, DISTANCE implies “the distance of each segment from the Jiyugaoka Station”. In the street choice model, it means “the distance from the destination”. If the respondents did not have their destinations defined, we set this value as “the mean distance from the station to every node in the study area”.

First, we explain PEDESTRIANS and CARS. A field survey was conducted five times on sunny weekday afternoons (14:00–15:00) in October 2012. We charted a survey route that did not overlap. Then, we traveled along that route on a bicycle while recording the route on a video camera (GoPro) mounted on our heads. The survey was conducted by travelling in two bicycles, covering a total distance of 10 km each day. After the survey, we counted the number of people and cars that we had bypassed on each segment. We divided the number of pedestrians by the area of the sidewalk, and calculated the logarithm of this value. This value corresponds to the variable “PEDESTRIAN”. Similarly, we divided the number of cars by the segment length and denoted the resulting value as “CARS”. PEDESTRIANS included people who were walking, stationary, and sitting. CARS included cars, bikes, and vehicles parked on the segment. Jiyugaoka station has two railway routes. Each route has both inbound and outbound lines, with trains arriving every 3 to 5 min on each line. The trains constantly arrive at the station, and hence we cannot observe apparent pedestrian waves diffusing from the station.

Next, we explain SHOPS. We obtained 1121 sets of shop data (retail shops and service shops) from Town Pages (Table 2). The parameter “school” includes small cram schools and cultural schools for adults.

**Table 2:** Number of each of the main shop-types.

Retail shops				Service shops					Total
Commodity	Fashion	General goods	Food	Restaurant	Café	Beauty salon	Beauty parlor	School	
44	244	131	77	268	35	92	123	107	1121

Then, we counted the number of shops facing the street and divided it by the segment length. Shops facing intersections were included in every segment. This value was called SHOPS.

Then, we explain ELEVATION. From 5 m DEM data by National Elevation Dataset, we got the closest points data of segments’ nodes and calculated the mean value of elevation between them. We used these values as ELEVATION of the segment.

**Figure 2:**
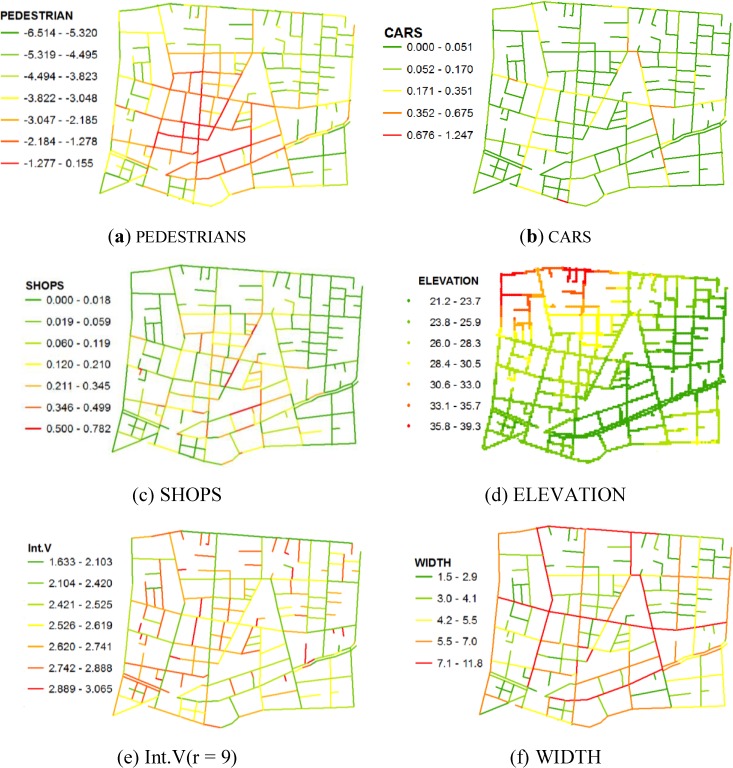
Values of each variable.

Then, we explain Int.V. We prepared an axial map that covers a 2-km radius from the Jiyugaoka Station. Then, we calculated the Axial Analysis (radius = 3, 5, 7, 9, n), Angular Analysis (radius = 1, 2, 3, 4, 5, n), and Segment Analysis (metric radius = 150, 300, 450, 600, 750, 900, 1050 m) by Depthmap. Figure 2 shows the values of these variables.

Finally, we explain WIDTH. We measured the street width in the study area using a walking measuring wheel. The widths of any sidewalks present were also measured.

## 3. Pedestrian Distribution Model

First, we evaluated the correlation between the predictor variables (Table 3). Then, we conducted multiple regression analysis using SHOPS, CARS, ELEVATION, DISTANCE, and each Int.V. The results differed with respect to the integration value (Table 4). As a result, the model employing Int.V (Axial Analysis radius = 9) showed the best result. Table 5 explains the details of the distribution model.

The model formula is

PEDESTRIANS = 0.923 × Int.V + 0.707 × SHOPS + 1.375 × CARS − 0.046 × ELEVATION − 0.002 × DISTANCE (from station) − 1.799
(1)

The multiple correlation coefficient was 0.728, and the contribution ratio was 0.530. The power of each predictor variable for the pedestrians’ distribution is in the order: DISTANCE > SHOPS > ELEVATION > Int.V > CARS.

We also considered other influencing factors such as CONNECTIVITY, CONTROL, and CHOICE, which are popular factors in space syntax analysis. However, these factors did not provide significant influence because their *p*-values were not appropriate. Although other factors may increase the accuracy of the model, we believe that the variables we used in the model are appropriate because they combine both street networks and other spatial compositions.

**Table 3:** Correlation between predictor variables.

	SHOPS	CARS	ELEVATION	DISTANCE
SHOPS		0.287 **	−0.060	−0.281 **
CARS			−0.035	0.770
ELEVATION				0.151 **

** *p* value < 0.05.

**Table 4:** Distribution models using each value of Int.V.

Int.V	R-value	R^2^-value	Adjusted R^2^-value	Standard error (SE)
Axial R3	0.719	0.518	0.504	0.403
Axial R5	0.722	0.521	0.508	0.402
Axial R7	0.725	0.525	0.512	0.400
Axial R9	0.728	0.530	0.517	0.398
Axial Rn	0.727	0.529	0.515	0.399
Angular_R1	0.707	0.500	0.487	0.410
Angular_R2	0.709	0.503	0.489	0.409
Angular_R3	0.710	0.505	0.491	0.409
Angular_R4	0.710	0.504	0.490	0.409
Angular_R5	0.710	0.504	0.490	0.409
Angular_Rn	0.709	0.503	0.490	0.409
Metric_150m	0.716	0.513	0.500	0.405
Metric_300m	0.722	0.521	0.508	0.402
Metric_450m	0.724	0.524	0.510	0.401
Metric_600m	0.717	0.514	0.501	0.404
Metric_750m	0.716	0.513	0.499	0.405
Metric_900m	0.712	0.508	0.494	0.407
Metric_1050m	0.707	0.500	0.486	0.411

**Table 5:** Details of the distribution model.

Explanatory variable	Non-Standardizing Coefficient		Standardizing Coefficient		*p*-value	Collinearity	
	Partial regression coefficient	Standard Error	Standardised partial regression coeficient	*t*-value		Tolerance	Variance Inflation Factor
Constant	−1.799	0.433		−4.158	0.000		
Int.V	0.923	0.269	0.192	3.426	0.001	0.829	1.206
SHOPS	0.707	0.128	0.311	5.538	0.000	0.825	1.212
CARS	1.375	0.853	0.089	1.612	0.109	0.854	1.171
ELEVATION	−0.046	0.010	−0.265	−4.835	0.000	0.862	1.160
DISTANCE	−0.002	0.000	−0.417	−7.476	0.000	0.832	1.201

## 4. Street Choice Model

### 4.1. Questionnaire Survey on the Visitors’ Strolling Route

We carried out a questionnaire survey to examine the routes taken by pedestrians in Jiyugaoka. Figure 1 shows the 14 points at which the survey was conducted. At each point, we interviewed five persons and obtained strolling routes of 70 persons. Table 6 shows the questionnaire items.

**Table 6:** Questionnaire items.

Items	Choices
Gender	Male, Female
Age	Teens, Twenties, Thirties, Forties, Fifties, Sixties, Other
Purpose	Shopping, Lunch, Rambling, Business, Get home, Other
Transportation mode	On foot, Bicycle, Bus, Train, Car
Travel time	< 30 min, 30 min, 1 h, 1.5 h, 2 h
Frequency	Once, Twice, Third times, Other,
Relationships	Friend, Parent, Couple, Other
Stationary time	Free answer
Route	Free answer

The pedestrians answered these questions and drew their routes on the A2-size map. We distinguished their street choices into the following categories: “headed toward their destination” and “having no set destination”. In this way, we used 46 persons’ routes (1211 street choices), which started from the Jiyugaoka Station. After this survey, we classified the respondents’ street choices by “toward destinations (TD)” or “non-destinations (ND)”, “male” or “female”, “couple”, “alone”, or “group”. Table 7 shows the number of street choices and the rambling ratio for each of these categories. The rambling ratio is the ratio of “the number of street choices for which destinations are not defined” to “the total number of street choices”. The rambling ratio of “female” is lower than other categories (“male” and “couple”). Also, the rambling ratio of “alone” is lower than that of “group”.

**Table 7:** Number of choices and rambling ratio for each attribute.

Attribution	Definition	Number of choices	Rambling ratio
All	All street choices	1211	34%
Toward destination (TD)	Heading for destination	799	0%
Non-destination (ND)	Undefined destinations	412	100%
Male	Only male (alone, group)	230	40%
Female	Only female (alone, group)	825	30%
Couple	Male and female group	156	46%
Alone	Street choices for alone person	129	30%
Group	Street choices for a group	780	36%

### 4.2. Logit Model

*V_ijn_* indicates the utility when an individual *n* chooses street *i* from *A_jn_* choices at the diverging point *j* during the trip chain. *A_jn_* is a set of streets from which an individual *j* choose one street at the diverging point *j*. “Utility” is the same as desirability or as benefit-cost index which a subject *i* receive. In this case, *V_ijn_* represents the sum of the products *X_ink_* (K pieces of elements) and constant *θ_k_* (parameter).

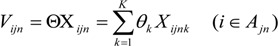
(2)


Θ = [*θ*_1_…*θ_K_*] is an unknown parameter vector, and *X_ijnk_* = [*X*_1_…*X_K_*] is a characteristic vector (choice *i* of individual *n* at diverging point *j*).

We assume that each individual consider both space connections and compositions and then choose a street to proceed. In this study, we set the representative utility function *V_ijn_* as follows:

*V_ijn_* = *θ*_1_*X_ijI_* + *θ*_3_*X_ijS_* + *θ*_4_*X_ijC_* + *θ*_5_*X_ijE_* + *θ*_2_*X_ijW_* + *θ*_6_*X_ijnD_* + *θ*_7_*X_ijnDirection_* (3)

Each predictor variable is defined as follows:

*X_ijI_*: Int.V (r = 9)

*X_ijS_*: SHOPS

*X_ijC_*: CARS

*X_ijE_*: ELEVATION

*X_ijW_*: WIDTH

*X_ijnD_*: DISTANCE (to the destination of individual *n*)

*X_ijn_*_Direction_: Direction (0 or 1)

*X_ijn_*_Direction_ is not the variable of space connection or composition. If the individuals choose to proceed straight along the forward direction, this value is set as 0. Otherwise, it is set as 1. We consider the tendency of the people to choose the straight direction on the basis of the previously reported study by Golledge [4,5].

Adopting random utility theory, logit model offers the probability function as formula (4). Formula (4) represents the probability that an individual *n* chooses subject *i* at diverging point *j*.

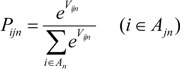
(4)


Next, we formulate the likelihood function. We consider the chosen results as *δ_ijn_*: *δ_ijn_* is 1 if individual n chose *i* at diversing point *j*, otherwise 0. Then, the joint probability, the likelihood *L* of individuals and their street choices is given as follows:

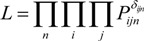
(5)


We find the values of parameter *θ_k_*, which maximize the likelihood function *L*, by Newton-Raphson method. This is a street choice model obtained by using the multinomial logit model.

### 4.3. Estimated Parameters for Each Attribute

We made eight logit models with respect to the attribute of strolling people and their destination; and estimated the best weight parameters for their street choices in each model. With the estimated parameter; we can see the weight of criteria which the strolling people value for their street choice. Table 8 shows the estimated parameters and hitting ratio (HR). Using the estimated parameters; probability of each choice can be estimated. The hitting ratio is the sum of the number of successes correctly predicted divided by the total number of cases when we predict strolling people will choose an option which has largest estimated probability. The hitting ratio is about 80%; and these models would be highly appropriate.

**Table 8:** Each estimated parameter and hitting ratio (HR).

Attribution	Int.V	SHOPS	CARS	ELEVATION	WIDTH	DISTANCE	DIRECTION	HR (%)
All	0.725	1.235 ***	−3.695	−0.160 **	0.104 ***	−0.052 ***	−1.354 ***	80
TD	−0.939	1.952 ***	−7.181	−0.262 **	0.132 ***	−0.534 ***	−1.238 ***	87
ND	2.010 **	0.582	−2.822	−0.122	0.071		−1.485 ***	67
Male	2.771 *	2.558 **	−12.929*	−0.148	0.265 ***	−0.067 ***	−1.635 ***	85
Female	0.565	1.155 **	−3.175	−0.060	0.095 **	−0.055 ***	−1.282 ***	80
Couple	0.283	0.957	3.363	−0.506 ***	0.038	−0.042 ***	−1.484 ***	77
Alone	1.881 *	2.411 ***	−15.222 **	−0.222 *	0.188 ***	−0.068 ***	−1.469 ***	84
Group	0.068	0.867 **	0.636	−0.128	0.074 *	−0.048 ***	−1.334 ***	78

* *p* value < 0.1; ** *p* value < 0.05; *** *p* value < 0.01.

If the *p*-value is lower than 0.1, we regard the variable as the element that influences the street choice. In all attributions, DISTANCE (from the destination) and DIRECTION produce a negative effect. Therefore, all people tend to proceed straight and choose streets that are near their destinations.

People who have defined their destinations (TD) tend to take streets that have many shops, and are wider and downhill. It can be assumed that their street choices strongly depend on the location of their destinations. Accordingly, it is important to consider the position and the composition of the attractive space to which many people tend to choose.

People whose destinations are undefined (ND) tend to take streets that have high Int.V. This implies that considering Int.V enables us to realize the city where people enjoy strolling even without purpose.

Males prefer streets that are wider, have higher Int.V, more shops, and fewer cars. Females prefer streets that are wider and have more shops. Couples tend to choose downhill streets. The street choice of males is affected by various elements. Or in other terms, males tend to choose their strategic route by considering various elements. In contrast, females and couples choose streets in a simple manner without much consideration. They are attracted by areas that have many shops and few rises.

Individual person tends to choose streets that are wider, downhill, and have higher Int.V, more shops, and fewer cars. On the other hand, people visiting as a group prefer streets that are wider and have more shops. The street choice of a person visiting alone is also affected by various factors. Therefore, they tend to choose streets by considering various elements like males.

At this point, although the distribution model revealed that pedestrian density is higher on streets with high car density, the route choice model indicates that pedestrians tend to avoid such conditions. This high pedestrian density may have been indicated because streets with many cars are important in street networks and in the strolling routes of pedestrians. However, because pedestrians are likely not comfortable in high-traffic areas, they tend to use other streets.

For ELEVATION, we conducted a questionnaire survey at nine points in the study area, which revealed that there are no confounders because of the route from a specific place. As shown Figure 2, the elevation of northwest and southeast are high, and that of southwest and northeast are low. It is implicated that these geographical features affect the street choice and that people tend to gather in basins because they avoid uphill walking.

## 5. Conclusions

In this study, we proposed a distribution model and a street choice model that considered both space connections and compositions. We developed a pedestrian distribution model that explained the characteristics of streets along which the density of pedestrians is high. It also clarified the power of each element. People are distributed on the streets that “are closer to the station”, “have more shops”, “are lower”, “have higher Int.V”, and “have more cars”. Although the distribution of pedestrians is less as being distant from the station, some spaces with high integration and shops have more pedestrians than other spaces. Therefore, it is highly imperative to carefully analyze the configuration of highly integrated space and shops.

Next, we analyzed the street choice of pedestrians strolling in shopping district by logit model and explained the differences in the trends of street choices on the basis of pedestrians’ attributes. For all the attributes considered in this study, the influences of DISTANCE and DIRECTION are strong. Hence, it is assumed that all people tend to choose streets that are straight and have the shortest path to their destination. The influences of Int.V, SHOPS, CARS, ELEVATION, and WIDTH are different for each attribute. It may be considered that these variables represent the difference in street choices made by different attributions. People with defined destinations tend to choose streets that “have more shops and are wider and lower”. In contrast, people without destinations tend to choose streets of high Int.V. The street choice of males is affected by Int.V, SHOPS, WIDTH (positive) and CARS (negative). Females prefer streets that have many shops and are wider, and couples tend to choose downhill streets. The choice of individual persons is affected by all variables. The behavior of people visiting in groups is affected by SHOP and WIDTH (positive).

These results correspond to the result of pedestrian distribution analysis. In order to realize a city that is more pleasurable for pedestrian walking, it is necessary to consider these characteristics of distribution and the mechanism underlining the street choice.

In future, we hope to conduct further studies in other cities and to conduct a more generalized analysis of the mechanisms related to persons’ walking patterns. Analysis in other towns and shopping areas would be necessary to attain deeper insights.
